# Pilot to policy: statewide dissemination and implementation of evidence-based treatment for traumatized youth

**DOI:** 10.1186/s12913-018-3395-0

**Published:** 2018-07-28

**Authors:** Lisa Amaya-Jackson, Dana Hagele, John Sideris, Donna Potter, Ernestine C. Briggs, Leila Keen, Robert A. Murphy, Shannon Dorsey, Vanessa Patchett, George S. Ake, Rebecca Socolar

**Affiliations:** 1 Duke University School of Medicine, 1121 W. Chapel Hill Street, Suite 100, Durham, NC 27701 USA; 2 The Center for Child and Family Health, 1121 W. Chapel Hill Street, Suite 100, Durham, NC 27701 USA; 3 University of Southern California, 1540 Alcazar Street, CHP 133, Los Angeles, CA 90089-9003 USA; 4 University of Washington, 335 Guthrie Hall, Box 351525, Seattle, WA 98195 USA; 5 Second Story, 2338 NW Overton St, Portland, OR 97210 USA

**Keywords:** Community implementation, Outcomes-oriented, Evidence-based treatment, Child trauma, Effectiveness, Learning collaborative, Implementation, Coaching

## Abstract

**Background:**

A model for statewide dissemination of evidence-based treatment (EBT) for traumatized youth was piloted and taken to scale across North Carolina (NC). This article describes the implementation platform developed, piloted, and evaluated by the NC Child Treatment Program to train agency providers in Trauma-Focused Cognitive Behavioral Therapy using the National Center for Child Traumatic Stress Learning Collaborative (LC) Model on Adoption & Implementation of EBTs. This type of LC incorporates adult learning principles to enhance clinical skills development as part of training and many key implementation science strategies while working with agencies and clinicians to implement and sustain the new practice.

**Methods:**

Clinicians (*n* = 124) from northeastern NC were enrolled in one of two TF-CBT LCs that lasted 12 months each. During the LC clinicians were expected to take at least two clients through TF-CBT treatment with fidelity and outcomes monitoring by trainers who offered consultation by phone and during trainings. Participating clinicians initiated treatment with 281 clients. The relationship of clinician and client characteristics to treatment fidelity and outcomes was examined using hierarchical linear regression.

**Results:**

One hundred eleven clinicians completed general training on trauma assessment batteries and TF-CBT. Sixty-five clinicians met all mastery and fidelity requirements to meet roster criteria. One hundred fifty-six (55%) clients had fidelity-monitored assessment and TF-CBT. Child externalizing, internalizing, and post-traumatic stress symptoms, as well as parent distress levels, decreased significantly with treatment fidelity moderating child PTSD outcomes. Since this pilot, 11 additional cohorts of TF-CBT providers have been trained to these roster criteria.

**Conclusion:**

Scaling up or outcomes-oriented implementation appears best accomplished when training incorporates: 1) practice-based learning, 2) fidelity coaching, 3) clinical assessment and outcomes-oriented treatment, 4) organizational skill-building to address barriers for agencies, and 5) linking clients to trained clinicians via an online provider roster. Demonstrating clinician performance and client outcomes in this pilot and subsequent cohorts led to legislative support for dissemination of a service array of EBTs by the NC Child Treatment Program.

## Background

With persisting disparities in mental health services for millions of children across the United States, evidence-based treatment (EBT) implementation and dissemination is essential to translational research designed to connect science and practice [1]. State and community agencies are partnering with mental health treatment developers and trainers in unparalleled numbers to bring effective treatments to underserved children and families. This is particularly evident in the field of child traumatic stress, in large part due to the Substance Abuse Mental Health Services Administration (SAMHSA) funded National Child Traumatic Stress Network (NCTSN), whose mission is to increase access to and quality of care in the US for traumatized children and their families.

Despite the increased emphasis on disseminating EBTs in community settings, without attention to the demands of service delivery and implementation, research indicates that an efficacious treatment model is no more likely to be delivered by clinicians than an inefficacious one [2, 3]. Successful adoption of any psychosocial treatment requires more than a workshop training approach to surmount clinicians’ wary attitudes toward manualized EBTs [3–5]. Data in the field of implementation science have demonstrated that the implementation process itself accounts for a significant proportion of the variance of treatment outcomes. Successful outcomes depend on our ability to develop effective strategies at various stages of implementation [6–10]. These strategies must address day-to-day challenges of assessment and service delivery, including: 1) barriers to client engagement [11]; 2) agency and practitioner implementation readiness [12–14]; 3) organizational culture and processes, including therapist turnover [15, 16]; 4) use of effective assessment that guides practice [17]; 5) mechanisms to address model fidelity [18–21]; 6) access to expert consultation during treatment of one or more cases [22–24]; and 7) application of quality improvement [25–28] and adult learning methods (i.e., interactive strategies that take into consideration previous knowledge and experience and appeal to different types of learners (visual, auditory, and kinesthetic) such as demonstrations, videos, group discussion, role-plays, and experiential activities) [29].

Improvement Collaboratives have emerged over the last decade to facilitate organizational change and EBT implementation. The collaboratives lead multiple organizations to apply quality improvement methods with the aim of closing the gap between potential and actual best practice [30–32]. The UCLA-Duke University National Center for Child Traumatic Stress (NCCTS) applied this methodology in 2005 to create a training/implementation model, the *NCCTS Learning Collaborative Model on the Adoption & Implementation of EBTs*, to better translate efficacious mental health treatments for child traumatic stress (CTS) into effective community clinical practice. Focusing equally on intense clinical training and enhancing implementation competence of a new EBT, the improvement goals included: 1) increasing the use of standardized assessments, 2) establishing clinician mastery of clinical skills and attaining model fidelity, 3) achieving expected clinical outcomes, and 4) addressing barriers to implementation and sustainability [33–35]. The NCCTS Learning Collaborative (LC) model has been used to maximize scalability and efficient use of the limited number of EBT developer-endorsed trainers. The NCCTS LC model is not only a training model incorporating important training tenets on teaching an EBT’s components and skills, but is designed to package and build capacity to integrate empirically established implementation science elements into the work with clinicians and their agencies across each phase of implementation [36] (Table 1). This means it is being used to train larger numbers of clinicians while also infusing implementation capacity that includes outcomes/improvement tracking. While there are select research teams looking to compare LCs to other forms of dissemination strategies [A. Herschell, personal communication, 8 October 2013; [36, 37], it is important to note that not all LCs are the same, enroll the same number of trainees, or use similar rigor in implementation and improvement science elements.

**Table 1:** Implementation Science Elements Addressed Within the NCCTS Learning Collaborative Model on Adoption & Implementation of EBTs (listed by EPIS^a^ Stages of Implementation)^b^

Exploration Phase	
1. Appropriate selection of EBT for population & gap in best practice [60]	
2. EBT format and training to fidelity can be replicated with multiple agencies [4, 5, 61, 62]	
3. Assessment of “readiness” for implementation: Appropriate selection of agencies based on implementation capacity [12, 13, 61]	
Preparation Phase	
4. Within selected agencies, selection of appropriate staff (defined team composition, including implementation champions) [13, 63]	
5. Attention to implementation process as part of variance of treatment outcomes [6–8, 10, 18, 64]	
6. Practitioner attitudes to EBTs [4, 65]	
7. Challenges to training within service delivery structure [66–68]	
8. Organizational readiness, culture, & processes addressed in preparedness & prework [16, 69]	
9. Data monitoring capacity at practitioner & agency level [70]	
10. Use of technology to integrate practice into care [71]	
Implementation Phase	
11. Multi-level agency-level organizational readiness to fully implement [72]	
12. Practitioner implementation readiness [73, 74]	
13. Recommended use of adult learning methods & behavioral rehearsal in training [75–77]	
14. Consideration of an appropriate coaching model in training and consultation calls [78]	
15. Day-to-day challenges of using assessment to guide practice [79]	
16. Day-to-day challenges to implementing a new treatment within service delivery structure [80–83]	
17. Model-specific client engagement [11, 84]	
18. Application of quality improvement as a practice change model [25, 26, 30, 31]	
Implementation Phase AND (Planning for) Sustainment Phase	
19. Mechanisms to assist & monitor model fidelity [18, 19, 21, 26, 85]	
20. Necessary capacity to use of data at the agency level [3, 86, 87]	
21. Applied use of metrics to assess and guide progress [31, 88, 89]	
22. Use of outcomes (clinical, functional, implementation) [17]	
23. External community stakeholders involved at key levels for referrals, community support & involvement in adoption & sustainment of EBT in community [90–92]	
24. Attend to barriers & facilitators of EBT’s sustainability prior to end of training & implementation [93]	
25. Involvement and support of senior leaders for facilitating agency decisions and navigating across leadership on behalf of EBT [92, 94–96]	
26. Therapist turnover during & after implementation process [72, 97]	
27. Strategies to assess clinician competence [98, 99]	
28. Model-specific supervision during & post training [5, 67, 100–102]	
29. Current & future use of EBT expert consultation & ongoing education for clinicians [103, 104]	

^a^Exploration, Preparation, Implementation and Sustainment Framework [2, 9]

^b^Table developed by Amaya-Jackson, Agosti, & NCCTS Training & Implementation Program (2014). v. 3/2018

### The North Carolina child treatment program

Even with the growing understanding of requisite components for successful implementation, limited guidance exists to identify and foster EBT readiness in community provider settings, to provide training and monitoring of fidelity, or to link children needing services to those EBT-trained professionals. The North Carolina Child Treatment Program (NC CTP) at the Center for Child & Family Health was created to address the quality of community mental health care for maltreated and traumatized children by combining state-of-the-art clinical training with public health principles and strategies gleaned from implementation science.

NC CTP was conceived as a training and treatment platform focused on dissemination and implementation of an efficacious treatment (Trauma-Focused Cognitive Behavioral Therapy [TF-CBT]) for sexually abused children experiencing post-traumatic stress [38–41]. Funders requested that the pilot focus on an underserved region of the state, thus it was conducted in a 28-county northeast region of North Carolina, comprising 15% of the state’s child population. Critical design elements included processes to: 1) recruit licensed clinicians who agreed to accept Medicaid reimbursement, 2) provide training and consultation to improve competence in trauma-specific assessment, TF-CBT, and implementation principles, 3) standardize fidelity and expected clinical outcomes necessary for certification and rostering, 4) maintain a public roster to link children and families with trained clinicians, 5) provide treatment funds for eligible, uninsured children, and 6) conduct a child and clinician outcome evaluation.

### Overview of the NC CTP pilot evaluation

The authors examined whether community-based clinicians working in rural, underserved geographic regions could apply an evidence-based trauma treatment with high fidelity through participation in a training and implementation LC. TF-CBT, the selected intervention, has been demonstrated as efficacious over the course of 15 randomized controlled trials (RCTs), yielding improvements in child and adolescent functioning across critical behavioral and emotional domains [38–42]. TF-CBT was selected because of its strong evidence base for improving PTSD, depression, and externalizing symptoms in children who have experienced sexual abuse and other traumas, along with its time-limited structure (12–20 sessions), offering a promising and affordable treatment for service delivery. TF-CBT has eight treatment components that are described using the acronym “PRACTICE”: Psychoeducation and Parenting skills; Relaxation; Affective expression and modulation; Cognitive coping; Trauma narrative exposure and processing; In vivo exposure; Conjoint sessions--where the child shares their trauma narrative with a supportive caregiver; and strategies to Enhance future safety and development. TF-CBT RCTs have been conducted across multiple trauma types (average number of trauma types in recent studies is 3.4), in multiple settings (residential, outpatient), and in the United States and low-resourced countries (e.g., 2 RCTs were done in the Democratic Republic of Congo for sexually trafficked girls and boy soldiers) [42].

There is little published on the use of learning collaboratives to promote adoption of mental health psychotherapies, though their use has increased in frequency as a dissemination tool for implementation-infused training. This evaluation of NC CTP’s pilot examines real world practice questions as to whether: 1) clinicians in a community practice setting could implement an EBT (e.g., TF-CBT) with a high level of practice fidelity through participation in a LC, and 2) youth who participate in a full course of TF-CBT provided by a clinician trained to model fidelity will experience clinically significant symptom improvements. Conducting this evaluation was a necessary step toward taking the program to scale. In order to move forward in seeking policy changes at the state level for dissemination of EBTs, community-level data were needed to prove program worth and feasibility.

## Methods/Design

### Participants

#### Clinicians

One hundred and twenty-four clinicians were enrolled in the first two TF-CBT LCs through the NC Child Treatment Program. These two training cohorts took place between September 2006 and March 2009 and provided the data for the pilot evaluation. Clinician eligibility criteria included: 1) possession of full or provisional NC licensure as a mental health clinician with training at a master’s level or above, 2) enrollment in Medicaid as a provider, 3) willingness to accept child protective services referrals and agreement to treat traumatized youth, including at least one sexually maltreated youth, 4) practicing in one of 28 identified counties, 5) having at least 50% of clientele ≤ age 17, and 6) agreement to submit client-level data for program evaluation. Clinician recruitment involved distribution of a brochure advertising free participation in a TF-CBT LC. Distribution targets included nine public mental health agencies, two Child Advocacy Centers, and the clinicians enrolled in the NC Medicaid program. Eligible respondents were enrolled according to the date their application was received.

Living in this rural, underserved region, the mental health clinicians were more likely than in the more populated areas of the state to contract with several agencies (rather than working full-time for a single agency), often working in several different counties. When asked upon enrollment about their prior 6 months of practice, 48% (*n* = 59) reported little to no treatment experience with traumatized youth. Clinicians were predominately female, had a Caucasian racial background, and were educated at a master’s level with a mean age of 45.7 years (see Table 2).

**Table 2:** Clinician and client demographics in pilot cohorts

Clinicians enrolled in cohort	Mean	Percentage	Total
			*N* = 124
Completed basic TF-CBT training		89.5%	111
Completed full rostering criteria (including monitored fidelity and outcomes) by study deadline		52.4%	65
Dropped out or failed to meet requirements		27.4%	34
Still in training at end of study		9.7%	12
Gender:		100%	124
Female		84.7%	105
Male		15.3%	19
Mean Age	45.7		
Race:			123^a^
African American/Black		18.7%	23
Multiracial		0.8%	1
White		79.7%	98
Other		0.8%	1
Ethnicity: Hispanic/Latino		2.4%	3
Licensure^b^			124
Master’s Level		91.9%	114
Nurse Practitioner		1.6%	2
Psychologist		10.5%	13
Trauma caseload before enrollment:			124
None		10.5%	13
Small (≤ 2 clients)		37.1%	46
Moderate (2–10)		25.8%	32
High (11–50)		26.6%	33
Clients enrolled in treatment			*N* = 281
Completed treatment with outcomes & fidelity monitored:		55.5%	156
With clinician who met fidelity standard		50.2%	141
With clinician not meeting fidelity standard		5.3%	15
Still in treatment after 3/31/09		12.1%	34
Exited early from treatment:		32.4%	91^c^
Client clinically unstable		3.2%	9
Home environment unstable		15.7%	44
Moved		6.4%	18
Transferred to another clinician		1.1%	3
Refused TF-CBT		2.5%	7
Other/unknown reason		9.3%	26
Gender:			279^a^
Female		77.12%	215
Male		22.9%	64
Mean Age	11.5		280^a^
Race:		100%	280^a, d^
African American/Black		11.0%	88 (279^a^)
American Indian/Alaskan Native		0.7%	2
Multiracial		6.4%	18
White		62.1%	174
Other		5.3%	15
Ethnicity: Hispanic/Latino		12.1%	34
Medicaid use		59.1%	163 (276^a^)
Sexual trauma reported at baseline		89.7%	252
Mean number of traumas at baseline	4.6		
Known contact with perpetrator during treatment		25.6%	72

*Note.* Percentages may equal greater than 100% due to categorical overlap

^a^Total scores in some categories vary due to missing data

^b^Some clinicians had multiple licensures

^c^Some clients exited early for more than one reason

^d^Some clients endorsed more than one category for race

#### Clients

Clinician-trainees assessed 281 child clients for treatment and study participation using measures listed below that documented their history of trauma and post-traumatic stress symptomatology. Exclusion criteria for a training case included: 1) acute suicidality, homicidality, or psychosis, or 2) absence of a caregiver willing to participate throughout the duration of treatment. Referrals were made to the clinician-trainee by local child welfare and mental health agencies, other community clinicians, court and school systems, or family members of the referred child.

### Training Design

#### Learning collaboratives

The project team adopted the *NCCTS Learning Collaborative Model on the Adoption & Implementation of EBTs* as the basis for the dissemination and implementation platform [33]. The NCCTS adapted the LC methodology for mental health EBTs from the Institute for Healthcare Improvement Breakthrough Series Collaborative (BSC), a quality improvement methodology for organizational practice change [30]. Unlike a traditional BSC, the NCCTS LC supplements the standard BSC’s focus on addressing organizational barriers with an additional intensive and experiential clinical skill-building focus toward competence in the chosen EBT. Agency leaders and clinical staff assess their organizational readiness for EBT-specific referrals, intakes, assessment protocols, metrics collection, and supervision practices and receive team time during the LCs face-to-face sessions to strategize on barriers internal and external to their clinics. Because rural North Carolina included large numbers of individual private practitioners in the pilot cohort, implementation strategies were applied to practitioners’ office practice as well as the usual application to agencies. The leadership of an LC must include designated experts in the selected EBT, in implementation in the child-serving community agencies, and in continuous-quality improvement methods. The trainers of TF-CBT in the LC must be developer-endorsed trainers. Essential LC components include: 1) three face-to-face Learning Sessions (2 days each) spread over a period of 9 months, covering clinical training, case-based learning activities, and skill-building in the EBT; 2) Action Periods (~ 2–3 months long) after each Learning Session are structured to facilitate clinicians’ application of learned skills with clients; 3) a secure intranet site to facilitate faculty-participant interactions and peer-to-peer learning, document the use of the model for improvement (12 calls), and plan-do-study-act cycles to address organization and system barriers; 4) group and bi-monthly (2× month) individual (1:1) fidelity consultation calls (coaching and monitoring) with a trainer on each component of each case; and 5) a senior leader track to guide agency administrative leaders in their support of implementation and change within the agency. As described above, many of the participating clinicians in the pilot cohorts were in solo practice, so the senior leader track had to be adapted to accommodate those private practitioners. Inclusion of private practitioners was less pronounced in later NC CTP training cohorts. Additional components of the LC include: 6) monthly metrics created in conjunction with the agency teams that monitor progress across agencies and practitioners—metrics such as the number of clients enrolled in TF-CBT; the number of sessions completed per client in TF-CBT, the number of clinicians treating a TF-CBT case; and 7) sustainability planning around modifications to participants’ practice that would be needed for future success in implementing the model beyond the LC. This would include session preparation and use of agendas, as well as, use of assessment measures to collect outcomes on every child. Particularly critical is building in model specific supervision and/or routine peer supervision focused on helping clinicians maintain TF-CBT fidelity in the context of case complexity.

An addition made specifically to the NC Child Treatment Program LCs is the individual (one-to-one) consultation calls (see #4 above) to monitor fidelity and provide case-specific coaching thereby augmenting the NCCTS LC Model usual reliance on group consultation calls for EBT coaching. These 1:1 (trainer-to-practitioner) calls were offered weekly in the first cohort and twice a month in the second cohort. During these calls, clinicians described what they did in sessions, and trainers monitored their fidelity closely via the use of the project’s Fidelity Consultation Metric (described below); trainers also coached clinicians on case treatment implications and planning/skill-building for the next therapy session. These calls, in addition to the group calls and the trainer/trainee discussion forums allowed the trainers a more complete picture of what was taking place in each therapy session.

#### Rostering and graduation criteria for clinicians

Successful graduation from an NC CTP LC required that clinicians: 1) complete TF-CBT*Web*, a 10-h, web-based overview of TF-CBT (www.musc.edu/tfcbt) [43], 2) attend an orientation and three (2 days each) face-to-face learning sessions, 3) participate in 14 clinical and implementation-focused group conference calls, 4) participate in all *individual* consultation calls with an assigned faculty member, 5) submit pre- and post-treatment standardized clinical measures for each enrolled client, 6) submit a clinical encounter summary following each session, and 7) complete at least one course of TF-CBT treatment with a sexually-traumatized child while demonstrating adequate fidelity. Upon completion of all requirements, trainees received 42 CEUs toward maintenance of their clinical license and were invited to join the publicly accessible TF-CBT “clinician roster” located on the NC CTP website. This requirement has since increased to completing monitored treatment of two clients, as discussed later in the paper.

### Measures used for clinical training and evaluation

#### Demographic variables

Gender, age, race, and ethnicity were collected from clinicians, who also reported this information about child clients and their caregivers. Additional clinician data included professional discipline, licensure, and practice experience. Additional client data included treatment completion status and history of sexual and other trauma (s).

#### Child measures

The assessment protocol collected information on history of trauma exposure, depressive symptoms, post-traumatic stress symptomatology, and general behavior problems. The costs of all measures were covered by NC CTP funds. *Trauma exposure* and *post-traumatic stress symptoms* were measured via the parent and child versions of the UCLA Post-Traumatic Stress Disorder Reaction Index (PTSD-RI) [44]. The PTSD-RI features 20 items that screen for exposure to traumatic events and 22 items that reflect the intrusive, avoidant, and arousal symptom clusters of PTSD, as well as total symptom severity. This measure is widely used for assessment of post-traumatic stress and has strong evidence for its reliability and validity. *Depressive symptoms* were assessed using the mean score on the Children’s Depression Inventory (CDI) Short Form [45], a 10-item self-report scale (with high correlation to the longer form [*r* = 0.89]) for youth ages 7–17. The CDI is a widely used measure with well-documented psychometrics. Two items were added to assess suicidality. Other aspects of *child behavior, social interactions, and functioning* were assessed with the Strengths and Difficulties Questionnaire (SDQ) [46]. The SDQ is a 25 item caregiver report form that assess an array of child behaviors, symptoms, and strengths. The SDQ is widely used in the US and global research; reliability (0.62), internal consistency (0.73), and validity are well established.

#### Caregiver measures

The Brief Symptom Inventory (BSI) [47] was used to gather information about the functioning of the child client’s parent/caregiver. The 18-item BSI addresses adult anxiety, depression, and somatic symptoms and provides a global index of symptom severity.

#### Clinician measures

Clinician fidelity and adherence to TF-CBT were assessed with the TF-CBT Fidelity and Clinical Competency Consultation Metric created for this project with the approval of treatment developers [48]. This instrument consists of 12 scales that allow a trainer to rate (on a 5-point Likert scale 0 = not addressed to 4 = addressed with fidelity and advanced clinical skill) each TF-CBT component applied by the clinician-trainee within a session. Clinicians did not do a self–report of fidelity. Rather fidelity and clinical competency in delivery of TF-CBT components, as rated by the trainers, were monitored during the consultation calls (weekly during year one of the pilot and twice a month in year two) and the clinician’s clinical encounter form (written). An overall fidelity score was determined by averaging the scores from each component. The 12 scales are: 1) pre-treatment assessment, 2) psycho-education, 3) parenting skills, 4) relaxation, 5) affective expression and modulation, 6) cognitive coping, 7) gradual exposure via trauma narrative, 8) cognitive processing, 9) in vivo desensitization, 10) enhancing future safety, 11) enhancing healthy development, and 12) post-treatment assessment and termination. Trainers routinely used role plays on calls and in learning sessions to train/assess fidelity and skill. There is evolving research supporting use of behavioral rehearsal as an analogue fidelity tool [49]. Clinicians were required to attain a mean fidelity and clinical competency score across scales ≥2.0 (Range 0–4.0). Inter-rater reliability was tested for 20% of faculty fidelity calls or 15 clinician-client dyads, yielding a concordance rate of 93%.

### Analyses

Data were collected as repeated measures prior to treatment and again upon completion. As some clinicians treated more than one child, the data were structured into three levels, with time nested within child and child nested within clinician. Analyses were run as hierarchical linear models with random slopes and intercepts [50]. Separate models were created for each of the child clinical outcomes, with each score converted to and analyzed as a *t*-score. Potential covariates (e.g., clinician age, race) were examined but not included in the final model if not significant.

## Results

### Training and performance results

At the end of data collection, 111 of 124 enrolled clinicians (90%) had completed the basic training on trauma assessment and the components of TF-CBT (taught in the early part of the collaborative). By the deadline for data collection, 65 clinicians (52% of the 124) met the full NC CTP rostering requirements (listed earlier), that included completing treatment with at least one child while demonstrating adequate fidelity. Another 12 clinicians (10% of the 124) fulfilled criteria after the deadline (determined based on time left on grant to complete data analysis) and were excluded from the research analyses as outcomes were not available in time. (Note: Eventual inclusion of these additional 12 resulted in a final tally of the program roster to be 77 (62%) clinicians from cohorts I and II). Thirty-four (27%) dropped out or failed to fulfill all requirements. Clinician-trainee reasons for dropout included failure to meet fidelity requirements (*n* = 6, 14%), clinician illness/death, departure from active practice, and inability to allot sufficient time from practice to meet requirements. Of the clinicians meeting fidelity on the TF-CBT Fidelity Consultation Metric (scoring between 2.0 and 4.0), the mode was 3.6 (range = 0–4). The curriculum allowed trainers to spend a lot of time with their trainees, with coaching/resource sharing on consultation calls, email, and internet blog. During the pilot, funding was sufficient such that clinicians could receive more extended clinical coaching and consultation for several months (~ 4 calls) beyond the year of bimonthly consultation offered in current cohorts. Clinicians who did not meet fidelity or program completion requirements but who were committed to trying to provide TF-CBT to children were invited to return to subsequent learning collaboratives.

When data collection stopped, 156 (55.5%) of the total 281 clients had completed treatment. One hundred and forty-one (50%) of the total 281 clients completed TF-CBT with a clinician who met the full fidelity and program requirements for rostering (see Tables 2 and 3). Fifteen (5%) clients had completed treatment under a clinician who did not meet fidelity standards.

**Table 3:** Client and Clinician Covariates used in Outcomes Analyses

Parameter Estimates	Total N (X %)
*Child/Client Covariates*	
Age, M (SD)	11.53 (3.85)
Gender, Female	279 (77%)
Race	
White	280 (62%)
Black	279 (11%)
Medicaid	276 (59%)
*Clinician Covariates*	
Fidelity (0–4), M (SD)	3.36 (0.64)
Prior knowledge of TF-CBT	281 (23%)
Psychologists	281 (9%)
Prior trauma caseload, M (SD)	10.20 (11.28)

Thirty-four clients (12%) were still in treatment at the end of the study, so outcomes were not yet available for analysis. Ninety-one (32%) clients discontinued treatment early with their primary three reasons being: 1) parent, client, or home environment was clinically unstable necessitating higher level of care, or 2) unsuitable for adhering to a training protocol, or 3) moved out of region or transferred care. Example situations of these cases that clinicians provided included clients running away, a change in group home status, parent crises (due to substance use or severe parental mental illness) interfering with treatment, and cases needing a new placement. Clients working with clinicians who dropped out of the program were not accessible for follow-up data. As expected, most clinicians ceased enrolling clients in CTP’s research data registry once they met their minimum rostering requirement, even though they then continued to treat youth using TF-CBT, and fulfilling their agency-mandated paperwork. However, of the 65 clinicians who completed rostering requirements, 20% completed additional monitoring in the 3–4 client category and 6% in the 5–8 client category in order to receive the (free) commensurate fidelity consultation.

### Clinical outcomes

All child clinical outcomes and caregiver distress outcomes decreased significantly from pre-treatment to post-treatment and are listed in Table 4 (all *p* < .001). Child depression scores decreased by almost one standard deviation (9.08, *p* < .001) as did suicidal ideation/intent (which was examined as a continuous variable). Mean scores for post-traumatic stress symptoms decreased significantly per both child (15.26, *p* < .001) and parent (10.23, *p* < .001) report, as well as for each symptom cluster (subscale). Caregivers reported a decrease in their own symptomatology as indicated by post-treatment BSI scores of approximately one half of a standard deviation (6.42) below pre-treatment levels. Caregivers also reported a total score symptom decrease for both the younger and teenage groups on the SDQ.

**Table 4:** TF-CBT Client Outcomes compared to Pretreatment Assessment on Clinical Measures

	Pretreatment	Posttreatment
	M (SD)	
Child Outcome Scores		
CDI^a^	54.35 (12.58)	45.28 (7.13)
Suicidal intent/ideation	1.35 (0.53)	1.18 (0.38)
Child^b^ PTSD total	33.62 (13.07)	18.36 (11.23)
Child: Reexperiencing	9.97 (5.36)	4.70 (4.08)
Child: Avoidance	12.08 (5.92)	5.99 (4.78)
Child: Hyperarousal	11.90 (4.28)	7.78 (4.10)
Parent^c^ Outcomes on Child		
Parent: Child PTSD total	28.97 (12.59)	18.74 (10.49)
Parent: Reexperiencing	8.30 (5.55)	5.37 (3.96)
Parent: Avoidance	9.77 (5.72)	5.99 (4.69)
Parent: Hyperarousal	11.00 (4.09)	7.42 (3.63)
SDQ 4–10^d^	17.25 (6.66)	11.82 (6.08)
SDQ 11–17^d^	18.41 (6.62)	12.54 (7.70)
Parent Outcome Scores		
BSI^e^	56.11 (12.41)	49.69 (10.51)

^a^*CDI* Children’s Depression Inventory

^b^Child refers to child’s response on the UCLA PTSD Reaction Index

^c^Parent refers to parent report of their child’s symptoms on the UCLA PTSD Reaction Index

^d^*SDQ* Strengths and Difficulties Questionnaire for ages 4–10 or 11–17, Total Difficulties

^e^*BSI* Brief Symptom Inventory, General Severity Index

Given significant main effects, tests for interaction effects for therapist and child characteristics (Table 5) followed. For the most part, therapist characteristics (e.g., prior TF-CBT knowledge, prior experience treating trauma clients) were unrelated to outcomes. Psychologist doctorates (vs. master’s level clinicians) had greater decreases in mean scores on younger clients’ SDQs, but did not differ significantly in any other domain. Among child characteristics, only child age was significant, and only for depression. Greater improvements in depression were evident among older youth.

**Table 5:** Regression outcomes controlling for child and clinician covariates^a^ with Fidelity Moderating Effects

Outcomes	Time	Fidelity	Time x fidelity
PTSD Child^b^	− 14.38 (1.05)***	−1.32 (1.71)	−3.84 (1.79)*
PTSD Parent^c^	− 8.98 (1.02)***	−0.29 (1.55)	−1.66 (1.65)
CDI^d^	− 8.41 (1.01)***	−0.43 (1.50)	1.80 (1.72)
SDQ4^e^	− 4.74 (0.81)***	−0.33 (1.41)	0.40 (1.48)
SDQ11^e^	− 6.08 (0.73)***	0.31 (1.20)	0.08 (1.11)

^a^Child covariates in the model were age, gender, race, Medicaid status; Clinician covariates were prior knowledge of TF-CBT, licensure status, and prior trauma caseload

^b^*PTSD Child* child’s response on UCLA PTSD Reaction Index (total score)

^c^*PTSD Paren* parent report on their child’s symptoms on UCLA PTSD Reaction Index (total score)

^d^*CDI* Children’s Depression Inventory

^e^*SDQ4 & SDQ11* Strengths and Difficulties Questionnaire for ages 4–10 or 11–17

*** (*p* < .001); *(*p* < .05)

**Fig. 1:**
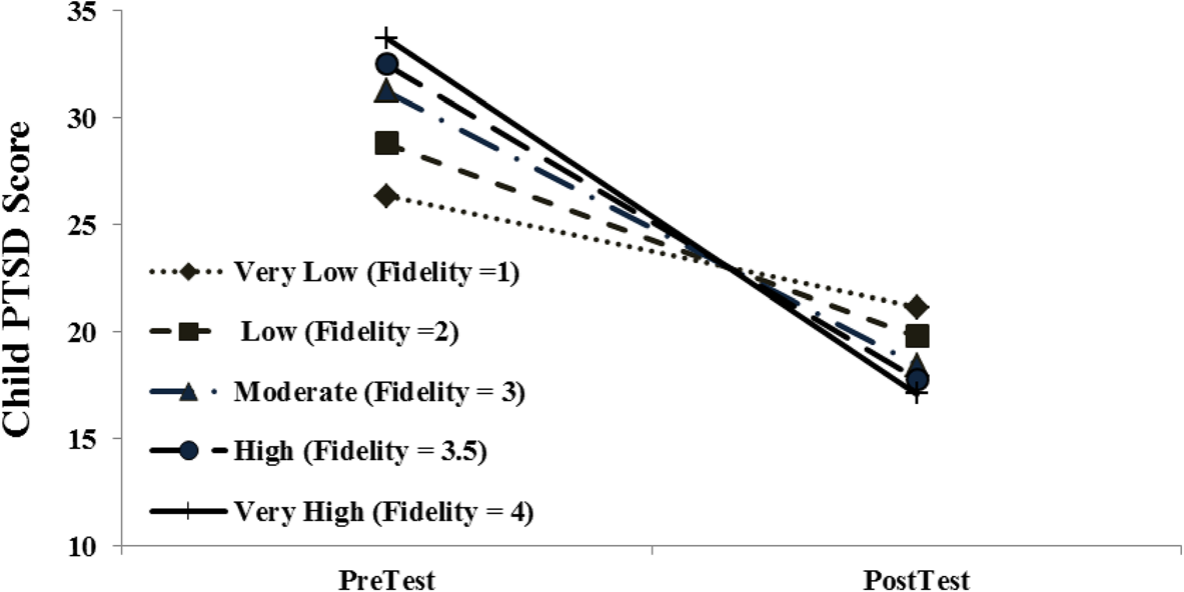
Fidelity-mediated child PTSD outcomes: Pre-treatment to Post-treatment

Subsequent to these models, we tested for the potential moderating influence of fidelity on primary outcomes (see Table 5). Fidelity significantly moderated one outcome, Child-Reported PTSD, F (1, 147 = 3.84, *p* < .05). The interaction is graphed in Fig. 1.

## Discussion

The challenge of bridging the “chasm” between research and practice requires not only training providers to demonstrate “clinical competence” in EBTs [51], but also addressing organizational and systems-level barriers that exist in delivering EBTs such as TF-CBT [3, 52–55]. This NC Child Treatment Program evaluation focused on whether (rural) community clinicians could be trained to fidelity in TF-CBT and effectively implement this EBT into their practice to achieve positive outcomes for their clients. The majority of clinicians were able to meet a rigorous fidelity competence threshold as monitored by the trainers. Equally important, they were able to learn and apply the use of assessment measures to their clinical work, develop an outcomes-oriented approach, and thereby document the effectiveness of treatment with their clients. Across key domains of child PTSD, depressive symptoms, including suicidal ideation/intent, and general mental health and behavioral difficulties, child clients improved significantly. Similarly, caregiver symptomatology improved. These outcomes were obtained while taking into consideration and controlling a number of clinician and client variables; not only client/clinician demographics, but clinicians’ prior clinical experience, training, and professional degrees.

While 90% of clinicians participated in the basic training on all components of TF-CBT, only 62% completed all requirements of coaching and monitored performance in taking at least 2 clients through treatment – and only 52% did so in time for their data to be included in the analyses. Beyond their reasons for drop out (illness/death; leaving practice; unable to get keep up with agency/practice demands while learning/applying a new model; failure to pass fidelity requirements) clinicians had to deal with the well-known problem in community mental health of client attrition; with 70% occurring after the first or second session [56]. 32% of these cohorts’ clients dropped out – hampering clinician’s ability to meet requirements if they did not have other trauma clients. The Northeast is underserved in terms of an adequate clinician base and agency resources. Given these regional concerns and the desire to train as many providers as possible, NC CTP applications to participate were less rigorous as they are now. Moreover, the senior leader track was not as well-developed as it currently is. Current clinician retention and rostering rates are 75 and 77% for the most recent completed 2 TF-CBT cohorts (see Table 6).

**Table 6:** NC CTP Learning Collaborative Enrollment: Pilot and Post-Pilot^a^

Cohorts	Clinicians trained		Clients^b^
	Enrolled	Rostered	
1 and 2	124	77	281
3 and 4	111	56	231
5 and 6	129	78	331
7 and 8	127	97	352
9 and 10	121	95	380
11	64	32	216
12	63	6	209
13	64	0	167

^a^The actual number of clients these clinicians have treated is many times the number of clients they enrolled for fidelity monitoring and is estimated to be well into the thousands. Additionally, all counties in NC are being served

^b^Enrolled by clinicians for monitored fidelity

There has been a surge in the use of the NCCTS Learning Collaborative model as a dissemination tool for training agencies wanting to adopt and implement EBTs, with more than 50 LCs administered by the NCCTS and the UCLA-Duke developers of LCs, as well as many other LCs conducted by other NCTSN sites. This evaluation adds to the growing literature on this model with outcomes and fidelity obtained at both clinician and client levels. The findings here show that community clinicians—even those providing treatment in rural regions known for having limited resources, when given the training and implementation supports necessary—can learn assessment administration and scoring, deliver TF-CBT with fidelity at high levels, and offer effective treatment for their clients as evidenced by their clients’ outcomes. These findings demonstrate remarkable promise of this training and implementation model.

A barrier that is documented in the literature—clinicians’ wary attitudes toward EBTs—proved unexpectedly to be less of an issue in this study, as evidenced by the fact that the first LC registration resulted in an immediate 200-clinician waiting list, despite its location in one of the largest underserved areas of the state. On the Evidence-Based Practice Attitude Scale [4] (used for training purposes only, not in the outcomes evaluation) administered to participating clinicians at the beginning of training, the primary barriers were not related to their attitudes about EBTs. Rather, the results showed clinician-identified barriers of access, cost of training, and the implementation hurdles in day-to-day practice; this confirmed the study team’s basis for developing NC CTP (to provide access to training) and using the LC model (to offer implementation solutions). Our impression was that community clinicians found this EBT acceptable and welcomed the type of training that directly applied the other aspects of evidence-based *practice* (integrating assessment, fidelity checks, clients’ needs/preferences) [51, 57]. Their candidness facilitated an appreciation of outcomes monitoring as being beneficial to clinical practice rather than only useful for research/evaluation purposes.

NC CTP’s attention to implementation barriers encountered in service delivery proved a key target of collaboration. Participants learned from each other as well as from faculty, sharing strategies with their fellow clinicians and agencies that, for example, increased referral volume, enhanced clinical coordination and care (e.g., seeing a child and parent in the same day); provided TF-CBT-specific documentation for Medicaid reimbursement, and improved their capacity to work with children who present with a number of systems barriers (e.g., foster care and juvenile justice placements). In LCs (generally), agency teams receive training in quality improvement methods to help test and refine strategies that will help improve practice [33]. Effective strategies are then shared across the collaborative to accelerate progress. Small tests of change, for example, a method of trying easily implemented changes and rapidly evaluating their utility and adjusting as necessary, developed in the NC CTP LCs include those that were used to improve waiting room environments, outreach materials, scripts to debrief parents on assessment measures, and session materials for young children receiving TF-CBT.

The expert consultation needed to address treatment integrity (fidelity) monitoring can be a costly clinical investment for any program. The fact that scrutiny and coaching to fidelity is an important focus for successful implementation was supported in our project by the relation between fidelity scores and decreased PTSD (measured by child report on the UCLA PTSD measure). The majority of clinicians were able to achieve high fidelity, a required component of the rostering process. Clinicians who struggled with low fidelity in this pilot and in subsequent cohorts were more likely to not have the pre-requisite clinical skills in client engagement, case conceptualization or basic CBT which may be related to their struggle with competency issues across components (e.g., cognitive coping, trauma narrative work and cognitive-affective processing) as similar findings were found by Hanson and colleagues [54]. Due to the pioneering nature of this pilot approach, we chose rigorous fidelity monitoring and coaching criteria (half an hour, twice a month for each clinician) not only to assist clinicians with their cases, but also to offer trainers valid information about session content in order to establish that the outcomes obtained were related to what clinicians were doing in session with their clients. There was little resistance from practitioners regarding the time we required they invest for coaching. The usefulness of the consultative coaching was such that participating clinicians were more likely to request additional consultation rather than resist calls. Interestingly, we found that with higher clinical complexity and client PTSD severity, fidelity levels were also the highest (see Fig. 1). This is likely explained by clinicians’ strong reliance on consultation when handling difficult cases. Over time, we plan to examine manpower requirements needed to document fidelity. However, given the success of this pilot and its subsequent cohorts that remain in very high demand (see Table 6), we have maintained this relatively high standard of consultation as criteria for all subsequent cohorts seeking to be rostered. Interestingly, attention to intensity and effectiveness of different consultation methods used in EBT training is now beginning to appear in research literature [22, 49, 58]. Maintaining the quality and sustainability of rostered clinicians’ practice through a post-training platform is now a major focus of our scope of work and includes advanced clinical trainings, webinars, role-plays of peer- supervision strategies and optional case consultation.

Key aspects of program development proved helpful in getting buy-in from stakeholders. Being able to demonstrate program effectiveness proved critical in order to obtain funding for future cohorts. In subsequent NC CTP LCs, tailoring the implementation curriculum to changing times and community differences was necessary (e.g., to help participants deal with mental health reform and changing state Medicaid rates; to work with communities with large numbers of military families; to deal with county differences in referrals and managed care entities) but was not resource-prohibitive. In the last 10 cohorts, we have increased the minimum requirement for treatment fidelity monitoring to two clients (one of which must be a sexually traumatized client) in order to broaden clinical experience and prepare clinicians for the forthcoming TF-CBT certification program created by the TF-CBT developers. Lastly, we created a public, web-based roster of the trained clinicians (www.ncchildtreatmentprogram.org) to provide referring professionals and families better access to highly-trained providers in their communities. The site allows a user to search by county to access a list of NC CTP-trained TF-CBT providers, their contact information, and the types of services they provide.

### Limitations

Given that the pilot cohorts took place in a rural area, the ability to generalize these training findings to other communities was a potential limitation. However, NC CTP has completed six additional TF-CBT cohorts across the state with similarly successful outcome results. This evaluation assessing and monitoring this training-implementation model was not a research controlled trial—making it possible that other factors influenced uptake and practice changes besides the participation in the LC program and NC CTP protocol. Pre- and post- assessments on clients as well as reports of session content were conducted by the trained clinicians themselves (to ensure the use of outcomes-oriented approaches in practice) - raising potential questions regarding measurement validity and possible clinician bias toward favorable reporting. However, scoring occurred in tandem with the trainers who were mailed the measures on each client, double-checked accuracy, and then addressed any additional questions regarding scoring on consultation calls. Similarly, each session’s content (12–20+ sessions/case) was reported in detail by clinician-trainees to their trainers using multiple venues (group calls, individual calls, and learning sessions). Children’s trauma narratives and parent sessions, were also used to identify inaccuracies in measurement or inconsistencies in reporting. Role-plays on calls and in learning sessions identified those trainees who were having difficulties with application of the treatment model components**.** The noted relationship between fidelity and child PTSD outcome must be taken with some caution as there was relatively low variance in fidelity scores; given the success of the trainers in building competence in the majority of clinician-trainees, very few clinicians had low fidelity scores.

## Conclusions

At the time of this writing, the NC CTP is conducting its 13th cohort of TF-CBT LCs. Pieced together with state child welfare grants, county funds, and tuition fees for cohorts three through eight, the CTP team had trained and rostered 305 clinicians in 92 of the 100 counties in North Carolina by 2012 and had built a prominent reputation based on clinician, agency, and parent consumer response. With generous time and support from key stakeholder groups, parent-consumer testimonials, education by program leadership, and advocacy efforts by the NC Child Fatality Task Force, NC’s General Assembly funded an annually-recurring state allocation for growth and sustainability of the program in 2013 to disseminate an array of five evidence-based treatments (including two LCs a year of TF-CBT) throughout the state of North Carolina using the described NC CTP LC and post-training platform. To date, as we complete cohorts 12 and 13 of TF-CBT dissemination efforts, iterative improvements in the NC CTP LC have occurred based on systematic use of participant feedback, agency metrics, clinician performance indices, client outcomes, implementation lessons learned, and use of a more sophisticated technology platform for innovative online data capture.

The NC CTP and its training protocol have successfully addressed what research has identified as four broad factors contributing to the gap between science and practice: 1) implementing high-quality service programs is complex, requiring significant knowledge and many skills; 2) individual clinicians must coordinate among different agencies, and communities must be ready to adopt and maintain new strategies; 3) financial, technical, and personnel resources are often insufficient; and 4) local clientele and circumstances may pose unique challenges for which there may be little guidance from research [59]. By emphasizing training in *implementation* as well as *clinical competence*, community clinicians were able to directly address these and other barriers to service delivery, and enhance skills in these same areas to address barriers they may encounter in the future. An unanticipated programmatic benefit was that promoting a senior leader (an agency administrator with the authority to enact agency level change) track in all subsequent cohorts created a network of administrative mental health community leaders. These leaders now wield valuable knowledge about the elements needed to build trauma-informed agencies and they carry this information into state and county stakeholder meetings that often have a strong influence on state and county policy.

## Abbreviations

BSI: Brief Symptom Inventory
CDI: Children’s Depression Inventory
EBT: Evidence-based Treatment
LC: Learning Collaborative
NC CTP: North Carolina Child Treatment Program
NCCTS: National Center for Child Traumatic Stress
NCTSN: National Child Traumatic Stress Network
PTSD: Post-Traumatic Stress Disorder
SDQ: Strengths and Difficulty Questionnaire
TF-CBT: Trauma-Focused Cognitive Behavioral Therapy
